# Scaling up stigma? The effects of antiretroviral roll-out on stigma and HIV testing. Early evidence from rural Tanzania

**DOI:** 10.1136/sti.2008.033183

**Published:** 2008-11-26

**Authors:** M Roura, M Urassa, J Busza, D Mbata, A Wringe, B Zaba

**Affiliations:** 1 Centre for Population Studies, Department of Epidemiology and Population Health, 49–51 Bedford Square, London School of Hygiene and Tropical Medicine, London, UK; 2 The TAZAMA Project, The Tanzanian National Institute of Medical Research, Mwanza, Tanzania

## Abstract

**Objective:**

To investigate the interplay between antiretroviral therapy (ART) scale-up, different types of stigma and Voluntary Counselling and Testing (VCT) uptake 2 years after the introduction of free ART in a rural ward of Tanzania.

**Methods:**

Qualitative study using in-depth interviews and group activities with a purposive sample of 91 community leaders, 77 ART clients and 16 health providers. Data were analysed for recurrent themes using NVIVO-7 software.

**Results:**

The complex interplay between ART, stigma and VCT in this setting is characterised by two powerful but opposing dynamics. The availability of effective treatment has transformed HIV into a manageable condition which is contributing to a reduction in self-stigma and is stimulating VCT uptake. However, this is counterbalanced by the persistence of blaming attitudes and emergence of new sources of stigma associated with ART provision. The general perception among community leaders was that as ART users regained health, they increasingly engaged in sexual relations and “spread the disease.” Fears were exacerbated because they were perceived to be very mobile and difficult to identify physically. Some leaders suggested giving ART recipients drugs “for impotence,” marking them “with a sign” and putting them “in isolation camps.” In this context, traditional beliefs about disease aetiology provided a less stigmatised explanation for HIV symptoms contributing to a situation of collective denial.

**Conclusion:**

Where anticipated stigma prevails, provision of antiretroviral drugs alone is unlikely to have sufficient impact on VCT uptake. Achieving widespread public health benefits of ART roll-out requires community-level interventions to ensure local acceptability of antiretroviral drugs.

WHO’s “3×5 strategy” was preceded by a wide debate about the feasibility and sustainability of antiretroviral therapy (ART) in resource poor settings, and potential synergies and conflicts between HIV treatment and prevention. Some argued that either treatment or prevention should be prioritised^1^ and warned about non-compliance and a potential increase in sexual risk behaviour,^2^ while others highlighted the limited efficacy of prevention-only programmes^3^ and assumed that ART roll-out would lead to a reduction in stigma by turning AIDS into a manageable condition. This would subsequently lead to improved rates of HIV testing^4^ ^5^ and safer sexual behaviours.

In 2002 UNAIDS declared “treatment and care for people with HIV/AIDS as fundamental to the AIDS response and its prevention,”^6^ and by 2003, WHO cited ART’s potential to reduce stigma and stimulate the uptake of VCT.^7^ Yet stigma continues to pose barriers to HIV testing.^8^^–^10

Some documented examples exist to suggest possible change. ART distribution initiatives in Haiti and South Africa, for instance, reported declines in stigma, increased HIV testing^11^ ^12^ and strengthened HIV prevention.^3^ While such experiences demonstrate the potential of treatment availability to dramatically reduce stigma and increase VCT uptake, “the global coverage of HIV testing remains unsatisfactorily low”^13^ despite progress made towards universal access. Recent studies in Sub-Saharan Africa (SSA) have further documented increased sexual risk taking after initiating ART^14^ and, for those testing negative, after undergoing VCT.^15^ The impact of ART provision on stigma reduction and VCT thus does not seem to be as clear as initially hoped. In Botswana, early provision of ART therapy did not encourage widespread testing, and HIV-related stigma persists.^16^ Similar findings have been reported in other SSA countries with generalised epidemics.^17^ ^18^

The relationship between ART and different forms of stigma is rarely addressed in empirical studies, despite well-conceptualised typologies of stigma existing in the literature.^19^

This qualitative study investigated the new dynamics between HIV stigma, ART provision and VCT uptake 2 years after the introduction of free antiretroviral (ARV) drugs in a rural ward of North Tanzania.

## Study location

This study was conducted in Kisesa, a ward located in North Tanzania where a monitoring project is documenting the implementation of the national ART programme within a cohort study established in 1994.

VCT services are available from a clinic in the trade centre and at temporary village-based facilities during periodical surveillance rounds. Referrals to the ART clinic located 20 km away in Mwanza city have been facilitated from VCT services in the ward since 2005. During the most recent surveillance round, 17.3% of the adult population agreed to undergo VCT in village-based mobile clinics, and routine data from the permanent clinic suggest that increases in VCT uptake during the first 2 years of the service have been slow.

**Box 1 Coding framework**

1. Types of stigma

- Discrimination
- Anticipated stigma
- Self-stigma
- Secondary stigma
- Multiple stigma

2. Sources of stigma

- Burden
- Fear
- Blame
- New sources
-
  - “They spread the disease”
  - Hungry/greedy

3. Attitudes

- Normalisation
- Diversity
- Contradictions
- By population groups (sex, age and income)
- Collective denial
-
  - “Kondela”
  - Blaming others (“Othering”)

## Theoretical framework

Drawing from “blaming models” and the conceptual work of Deacon, we understood stigma as “an emotional response to danger that makes people feel safer by projecting controllable risk, and therefore blame, onto out-groups.”^19^ This process creates boundaries between the “normal” and those who are “discredited within a local moralistic system”^20^ and *may* be used as an instrument to maintain the “social order.”^19^ ^21^ HIV stigma would then be based on both “disease avoidance” and “norm enforcement.”^22^

## METHODS

Semistructured interviews and group activities based on visual outputs were conducted with 91 community leaders, 77 ART clients and 16 health providers (table 1). Theoretical sampling was used, where participants continue to be recruited until no new information emerges.

**Table 1:** Characteristics of the study sample (n = 184)

	Trade centre	Rural areas	Total
Community leaders	33	58	91
Formally elected			
Village leaders	4	6	10
Ten-cell and subvillage leaders	18	40	58
Informal			
Faith leaders	3	4	7
Village advisers (“maarufu”)	6	6	12
Traditional healers	2	2	4
Persons living with HIV/AIDS	39	38	77
Male	20	18	38
Female	19	20	39
Health providers	9	7	16
Facility-based	7	4	11
Community-based	2	3	5
Total			184

With the aid of six resident fieldworkers involved in the cohort study, all the village-level leaders of the ward were invited to participate, and a sample of subvillage and ten-cell leaders was purposefully selected to ensure balanced geographical representation.

Influential individuals often consulted for advice (“Maarufu”) and other informal leaders were identified through snowball techniques whereby sampled individuals suggested additional participants through mapping exercises. Building consensus within the group during the identification of influential persons was a core component of the activity in order to prevent informants from directing us towards closely related villagers likely to hold similar opinions.

Persons living with HIV/AIDS (PLHA) were purposively sampled to ensure the distribution across sex, age and area of residence reflected that of all referrals made by the VCT centre. Invitations were delivered through the VCT counsellors and during monthly post-test club meetings. Both individual interviews and group discussions were conducted in order to identify any potential reporting bias.

The fieldwork guidelines consisted of open-ended questions without explicit references to stigma. The iterative approach adopted—in which data collection and analysis take place simultaneously—allowed for ongoing refining of field tools as relevant topics emerged.

Informed consent was obtained from all participants, and verbal data were tape-recorded, transcribed and translated into English. Fieldworker notes provided non-verbal information and pictures of visual outputs facilitated analysis and cross-case comparison.

The software package NVIVO 7 was used to identify major themes. The first author analysed 15 sample transcripts to develop a coding frame centred on the main types and sources of stigma (table 2). This was amended throughout coding (box 1). A second researcher independently coded a random selection of data in the original language. Emerging hypotheses were compared and tested by re-reading transcripts and fine-tuning interpretations.

**Table 2:** Different types and sources of HIV stigma

Types of stigma	
Discrimination	Enacted stigma; what people do to unfairly disadvantage known or suspected HIV positive persons, such as exclusion from shared activities
Anticipated stigma	The stigma people expect from others if they were known to be HIV positive
Self-stigma	Internalised feelings of shame or blame derived from accepting stigmatising judgements of one’s identity
Secondary stigma	Stigma which, by association, affects those related to the infected
Sources of stigma	
Burden	Stigma derived from the inability of individuals to conduct productive activities and look after themselves, leading them to be perceived as a “drain of resources”
Fear	Stigma derived from the fear of being infected by HIV through casual or sexual contact
Blame	Stigma derived from the association of HIV with negatively defined behaviours or groups in society, which are subsequently blamed for their infection

Source: Based on Deacon H (2005) *Understanding HIV/AIDS stigma. A theoretical and methodological analysis*. Research Monograph. London: HSRC Press, 2005.

Ethical approval was obtained from the LSHTM Ethics Committee and the Tanzanian Medical Research Coordinating Committee.

## RESULTS

### Towards a normalisation of HIV: “It has become the same as fever”

Burden-related stigma, focused around the inability of PLHA to conduct routine activities, emerged as a major source of HIV stigma in this community. As ART visibly restored normalcy in clients’ productive lives, this source of stigma appeared to decrease, as did PLHA’s own feelings of shame. Many felt comforted that the disease had become “just like malaria” and mentioned feeling “like a normal person.” They reported realising that the disease was “for everyone,” including “innocent” and “respectable” people, through interaction with health professionals and other PLHA at the ART clinic.

I just felt good because this disease has now become just as any other disease…she told me that I should be calm: “you just get treatment, this has become just like fever”… (ART user, trade centre, female)

This catastrophe is not for me alone…it can affect anyone. (ART user, remote rural, male)

ART clients were successfully encouraging others to seek treatment, and a few reported that they had been tested after being advised by a person already on antiretrovirals (ARVs).

… they are going in front telling: “I am HIV positive…I am OK, so come and check your health so you can start ART early before becoming sick”… (healthcare provider, female)

### Pervasive blame: “All of this because of a two minutes pleasure”

Most community leaders interviewed attributed HIV infection to personal decisions over sex life and alcohol consumption. Such behaviours were perceived as degrading and avoidable, and PLHA were consequently accused of being “negligent,” “irresponsible” and “lazy.” Greater blame was placed on those who consumed alcohol, and if they experienced treatment failure, this was assumed to be due to sexual activity while on ARVs, and thus a “deserved” outcome of unacceptable behaviour.

…they die because of negligence. Even after you die they will say: “this old man has caused problems to his children. If he was alive his children wouldn’t be in this situation, but all this is because of a two minutes pleasure”… (VL9, semi rural, male)

Others have used them (ARVs) wrongly. God was pleased to take them early…but all of them were using the drugs and still having sex with other women. (VL3, trade centre, male)

This disease is transmitted through immorality. (TH1, remote rural, female)

Women were sometimes accused of “provoking men” but could be exempted from blame due to poverty and powerlessness.

Respondents credited HIV education with decreasing local stigma. However, the impact seemed superficial, considering the wide heterogeneity of attitudes towards PLHA, the frequency of contradictory statements and the persistence of anticipated stigma as a leading barrier to VCT uptake.

Some are sad and others are happy when someone is sick because maybe s/he was very promiscuous. Now s/he has been caught. (VL2, semi rural, male)

Youth find it difficult: “If they see me there (VCT centre) I will lose girlfriends, I will lose money”… (VA1, trade centre, male)

### Scaling up stigma? “Let him take these medicines, but he should be made impotent”

The majority of community leaders interviewed expressed deep worries about a potential increase in HIV incidence as a direct consequence of ART roll-out. This concern was also raised without any prompting by members of Aids Committees in the five villages where group discussions took place. The general perception among both formal and informal leaders and across groups of sex and area of residence was that as PLHA regained weight and health, they were increasingly engaging in sexual relations and “spreading the disease.” In exceptional cases, participants believed that PLHA had reduced the frequency of sex because they were being “avoided by others.” However, in general terms, fears surrounded the belief that PLHA on ART looked “attractive” and could not be physically identified. Furthermore, they were perceived to “live longer” and “move around,” giving additional opportunity to spread the disease. Other concerns were that HIV drugs caused mental disorders, “aggressiveness,” “gluttony” and “greed.” In a context of extreme poverty, the economic and nutritional aid provided to ART clients through support programmes was not always appreciated by other community members. Some village leaders suggested public disclosure of HIV test results, giving ART recipients drugs “to make them impotent,” marking them with a sign and putting them “in isolation camps” (box 2).

**Box 2 Scaling up stigma?**

- These antiretrovirals are not useful…they make him look healthy so if you don’t know him you may have sex with him and then you are infected too. We advise the government and relevant organisations that once someone is discovered to be HIV positive he should be given medicine to make him impotent. Let him take these medicines and become fat but he should be made impotent so that he should not look for women anymore. (VL1, semirural, male)
- …when s/he will see that his health is good s/he will continue spreading infections…The government should mark them so we can know who is infected…a certain region for those people with HIV would be safe. (VL10, remote rural, male)
- …people are getting drugs and their conditions become well…someone can go to have sex with them, they think that this person is well…others are even getting married…I think the disease will spread because of those drugs. (VA2, trade centre, female)
- ….they are continuing bearing children. If the community has three families of that type many children will be sick. (Group activity 2, remote rural, males and females)
- they start saying that these people are spreading AIDS. They are just going to get AIDS drugs. Now we live just like that… without testing… (VA4, remote rural, female)

### Collective denial: “AIDS doesn’t exist. We just bewitch each other”

The belief that a wide array of diseases can be caused by witches as a consequence of jealousy or revenge is widespread in this community. Some community leaders referred to a disease from the past (“Kondela”), which looked like AIDS, was caused by witches and could be cured by Traditional Healers specialised in “divination.”

In the past the Sukuma called it “Kondela.” A person slims and looks like s/he is HIV positive, but s/he is not. It’s a disease similar to AIDS caused by medicines of the witches. It is often used if you quarrel with someone… if s/he has got that medicine s/he bewitches you. (VA1, trade centre, male)

Contrary to PLHA, people suffering from “Kondela” were not feared or blamed for a condition which was perceived to have been caused “to” them and not “by” them. This contributed to a situation of collective denial with a negative effect on VCT uptake.

Their relationship with him/her is good because they believe that s/he is bewitched… because they don’t really believe that this person has AIDS. If they really knew that this person has AIDS it’s tough in the village. S/he is not taken care of. They say: “s/he wanted it, s/he liked it.” They know that s/he got it through sex. (VA7, semi rural, female)

They had false beliefs that perhaps s/he is bewitched…s/he says: “I have no disease, they are lying, they didn’t check it well…” (healthcare provider 4, female)

if s/he dies…one group will say it’s AIDS… but his/her close relatives will oppose it: “it wasn’t AIDS, it was “this and this” (witchcraft) …s/he has passed away on unidentified diseases…and s/he wasn’t even checked. (VL5, trade centre, male)

## DISCUSSION

**Figure 1:**
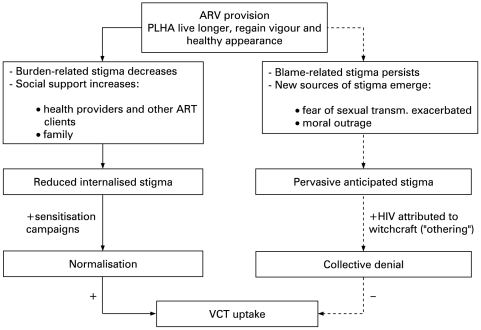
Effect of antiretroviral (ARV) provision on HIV stigma and Voluntary Counselling and Testing (VCT) uptake: early evidence from rural Tanzania. ART, antiretroviral therapy; PLHA, persons living with HIV/AIDS.

Our findings cast doubts on the assumption that ARV provision will lead to a reduction in stigma and a substantial increase in VCT uptake. Instead, two seemingly opposing dynamics emerge. On the one hand, the availability of effective treatment has transformed HIV into a chronic, invisible and manageable condition which is contributing to a reduction in self-stigma and growing openness among ART clients. Such “normalisation” of the disease can encourage VCT uptake. On the other hand, the persistence of blaming attitudes in the community and the emergence of new sources of stigma directly associated with ARV provision fuel stubbornly high levels of anticipated stigma, which contribute to disease denial and discourage VCT uptake (fig 1). These counterbalancing trends are likely to explain the relatively slow increase in the rate of VCT uptake, and context-specific manifestation of each type of stigma will need to be considered in order to develop appropriate interventions.

**Key messages**

- There is insufficient evidence for the common assumption that antiretroviral therapy (ART) provision automatically leads to reduction in stigma and increased HIV test uptake.
- In our study setting, the complex interplay between ART, HIV stigma and Voluntary Counselling and Testing (VCT) is characterised by two powerful dynamics in different directions.
- The positive impact of HIV normalisation on VCT uptake is counterbalanced by persistent blaming attitudes and the emergence of new sources of stigma.
- Achieving widespread public health benefits of ART roll-out requires community-level interventions to ensure local acceptability of antiretroviral drugs.

Where PLHA are blamed for their condition, identified as a modern, sexually transmitted disease, and belief in supernatural forces as the origin of a wide array of diseases prevails, there might be further incentives to avoid testing and the risk of receiving a clear HIV diagnosis. As some cases of witchcraft are not subject to social sanction, cultural beliefs about disease aetiology provide a mechanism to blame others and turn the “guilty” into “victims,” thus allowing widespread avoidance of confronting the epidemic.

Targeted interventions may be required to prevent a scale-up of new types of stigma, tackle the deeply rooted association of HIV with “immoral behaviour,” and ensure the local acceptability of ART. Community leaders, opinion makers and representatives of community-based organisations should be made aware of the adverse effects of stigma, and home-based care providers should continue to act as “role models.” Positive prevention messages addressed to ART clients covering all available options should be delivered at health facilities and community settings, and the effects of prioritising PLHA when delivering food support within impoverished communities considered. Opinion leaders able to influence norms and values should closely collaborate with PLHA in community sensitisation activities promoting VCT uptake and emphasising that HIV can affect anyone.

The proposed interventions are backed by previous initiatives demonstrating that education interventions addressed to ART clients can contribute to safer sexual behaviours^23^ and that existing community assets can be mobilised to tackle stigma and promote HV testing.^24^^–^27 However, uncertainties remain about the overall impact of ART provision and VCT on sexual behaviour^15^ ^28^ ^29^ as well as the long-term effect of ART provision on stigma and VCT uptake.

Our findings are constrained by some methodological limitations including reliance on self-reported data and use of snowball sampling. PLHA might have under-reported negative experiences, and community leaders could have directed us to persons holding similar opinions. However, the sampling of key informants was conducted through group exercises, and we triangulated information by using different data-collection tools and categories of participants.

The challenges we have identified have been documented in other areas of Tanzania^30^ and are likely to be similar to those faced in rural SSA settings where ART provision is being scaled up through National AIDS Control Programmes.

## CONCLUSION

The tendency towards a normalisation of HIV resulting from the availability of ART provides a unique opportunity to maximise the synergies between HIV treatment and prevention. However, this opportunity might be lost in the absence of vigorous community mobilisation interventions to ensure local acceptability of ARV drugs.
